# Correction to: Administrative Claims Data Versus Augmented Pregnancy Data for the Study of Pharmaceutical Treatments in Pregnancy

**DOI:** 10.1007/s40471-018-0132-5

**Published:** 2018-01-23

**Authors:** Susan E. Andrade, Anick Bérard, Hedvig M. E. Nordeng, Mollie E. Wood, Marleen M. H. J. van Gelder, Sengwee Toh

**Affiliations:** 1 Meyers Primary Care Institute, Fallon Community Health Plan, Reliant Medical Group, University of Massachusetts Medical School, 425 North Lake Avenue, Worcester, MA 01605 USA; 2 Faculty of Pharmacy, and CHU Ste-Justine Research Center, University of Montreal, 3175 Côte-Ste-Catherine, Montreal, QC H3T 1C5 Canada; 3 Pharmacoepidemiology and Drug Safety Research Group, School of Pharmacy, PharmaTox Strategic Research Initiative, Faculty of Mathematics and Natural Sciences, University of Oslo, P.O. Box 1068, Blindern, 0316 Oslo, Norway; 4 Department of Child Health, Norwegian Institute of Public Health, P.O. Box 4404, Nydalen, 0403 Oslo, Norway; 5 Department for Health Evidence, Radboud Institute for Health Sciences, Radboud University Medical Center, P.O. Box 9101, 6500 HB Nijmegen, The Netherlands; 6 Radboud REshape Innovation Center, Radboud University Medical Center, P.O. Box 9101, 6500 HB Nijmegen, The Netherlands; 7 Department of Population Medicine, Harvard Medical School and Harvard Pilgrim Health Care Institute, 401 Park Drive, Suite 401 East, Boston, MA 02215 USA

**Correction to: Curr Epidemiol Rep (2017) 4:106–116**

10.1007/s40471-017-0104-1

The article “Administrative Claims Data Versus Augmented Pregnancy Data for the Study of Pharmaceutical Treatments in Pregnancy,” written by Susan E. Andrade, Anick Bérard, Hedvig M.E. Nordeng, Mollie E. Wood, Marleen M.H.J. van Gelder, and Sengwee Toh, was originally published Online First without open access. After publication in volume 4, issue 2, and pages 106–116, the author decided to opt for Open Choice and to make the article an open access publication. Therefore, the copyright of the article has been changed to © The Author(s) 2018 and the article is forthwith distributed under the terms of the Creative Commons Attribution 4.0 International License (http://creativecommons.org/licenses/by/4.0/), which permits use, duplication, adaptation, distribution, and reproduction in any medium or format, as long as you give appropriate credit to the original author(s) and the source, provide a link to the Creative Commons license, and indicate if changes were made.

